# An Improved Graph Model for Conflict Resolution Based on Option Prioritization and Its Application

**DOI:** 10.3390/ijerph14111311

**Published:** 2017-10-27

**Authors:** Kedong Yin, Li Yu, Xuemei Li

**Affiliations:** 1 School of Economics, Ocean University of China, Qingdao 266100, China; yinkedong@ouc.edu.cn (K.Y.); yuli920718@163.com (L.Y.); 2 Ocean Development Research Institute, Major Research Base of Humanities and Social Sciences of Ministry of Education, Ocean University of China, Qingdao 266100, China

**Keywords:** graph model for conflict resolution, sustainable development on conflict analysis, option prioritization, real-coalition stability analysis

## Abstract

In order to quantitatively depict differences regarding the preferences of decision makers for different states, a score function is proposed. As a foundation, coalition motivation and real-coalition analysis are discussed when external circumstance or opportunity costs are considering. On the basis of a confidence-level function, we establish the score function using a “preference tree”. We not only measure the preference for each state, but we also build a collation improvement function to measure coalition motivation and to construct a coordinate system in which to analyze real-coalition stability. All of these developments enhance the applicability of the graph model for conflict resolution (GMCR). Finally, an improved GMCR is applied in the “Changzhou Conflict” to demonstrate how it can be conveniently utilized in practice.

## 1. Introduction and Motivation

Conflict analysis is a branch of game theory, and the graph model is an important method for solving conflict. The basic idea is to use related knowledge of subsets and graph theory to obtain an equilibrium state. Fraser and Hipel simplified metagame analysis and developed it into published work on F-H conflict analysis [1,2]. Later, Kilgour et al. continued this work and evolved it into the graph model for conflict resolution (GMCR) [3], which they described comprehensively and minutely. 

Since it was first proposed, scholars have studied GMCR from multiple perspectives: decision makers (DMs) who hold different attitudes and emotions would have different effects on the results of conflict. Walker et al. used the attitude of DMs as a foundation on which to construct four preferences and four stabilities [4]. Obeidi et al. considered emotion as an essential modifier in conflict analysis, and used the example of U.S.–North Korea conflict to demonstrate that the graph model could be simplified after taking emotion into account [5]. Research on the preferences of DMs generally considers four aspects: fuzzy preferences, gray preferences, strength of preferences, and unknown preferences. Mubarak et al. proposed an improved method for fuzzy preference that was based on DMs holding five different preference degrees on feasible states. Furthermore, they quantified the preference degree for each DM through piecewise functions and redefined the four stabilities. They then resolved the conflict and achieved an equilibrium state [6]. Option prioritization was first proposed by Wang and Hipel [7]. Thereafter, Bashar put forward the definition of fuzzy truth value and improved option prioritization [8]. Yu and Yu researched fuzzy preference further, constructing a framework for calculating fuzzy preferences of DMs and applying it to practical conflict [9]. Kuang et al. [10] determined the preference sequence by using a gray-based preference structure and redefined gray stabilities. They then applied the improved model to a brownfield conflict in Ontario, Canada. The interval gray number was used by Yang and Guan to determine preference ranking and was applied to industry–university cooperation conflict [11]. Liu continued an intensive study into the combination of gray number and GMCR to solve equilibrium states and decision paths [12]. Hamouda et al. [13] extended DM preference to a triplet preference structure. They generalized the basic concept on a subset and then refined strong and weak stability. Finally, the strength of preference was applied to a water conflict. 

Kevin and Hipel considered uncertain preference as a potential improvement. They redefined the formal definition and enhanced the applicability of the graph model by proposing an algorithm to generate a status quo diagram and table. They then analyzed an environmental conflict to demonstrate how the new algorithm could be applied [14]. Yu and Hipel redefined two logical connectives to determine an unknown preference, and applied it to a water dispute [15]. With respect to coalition analysis, Kilgour et al. depicted the fundamental definitions of coalition analysis in detail, and illustrated the procedure in GMCR II with a simple model [16]. Kinsara and Kilgour introduced third-party intervention to GMCR and constructed inverse GMCR to measure how third parties influence the process of conflict [17]. Kinsara and Hipel preferred three approaches to improve and simplify the understanding of conflict results [18].

The process of solving GMCR is concise, and the procedure is clear, so it is widely used in negotiation support, water resource protection, brownfields, complex products, sustainable development, energy, and other fields. Hipel and Kilgour used a Canadian groundwater storage conflict as an example, elaborating the application of GMCR in solving environmental conflict in detail [19]. Karnis et al. used GMCR to analyze the conflict in relation to the international upper great lakes [20]. Huang and Liu expounded the definition of seven types of stability and expanded the application of GMCR to business negotiations [21]. Wang and Kilgour put forward a risky project negotiation framework to measure market risk and provide a resolution of conflict [22]. Kuang and Bashar constructed a gray preference to express DM preference and used it to resolve conflict over brownfield redevelopment [23]. Han and Xu extended the application of the graph model to the complex process of aerospace products. In addition, they reconsidered the equilibrium state of simple preferences based on the economic and corporation-reputation orientation of DMs [24]. Chu and Hipel provided a win-win resolution for the Zhanghe River water-allocation dispute in China through GMCR [25]. Li and Han made use of Matrix Representation of the Solution Concept (MRSC), a decision-making system, to analyze credit expansion and concentration based on GMCR [26].

Currently, in the decision-support system GMCR II, there are three simple preference-ordering methods for feasible states: direct rank, option weight, and option prioritization. For option prioritization, each DM’s preference statements are listed in order of priority, often represented vertically from the most important to least [27]. Option prioritization is a very useful preference modeling technique and overcomes the limitations of other methods [26]; however, option prioritization also has its shortcomings. In the vast majority of real-life situations, DMs cannot grasp all the information, or also cannot hold a clear attitude on the issue of conflict, so when DMs express their preference information, they usually do not have full confidence in every preference statement. For instance, DMs definitely ensure the preference statements that are considered initially. As the preference statements are represented one by one from the most important to the least important, the DMs start to doubt the order and validity of preference statements, and this uncertainty will gradually increase in the process of establishing preference statements. A higher confidence degree of a preference statement means higher suitability, while a lower confidence degree of a preference statement indicates a lower suitability. While this uncertainty in preference statements will affect preference difference between different states. For two different states, the preference difference may be either extremely small or severely large. Moreover, the uncertainty will affect coalition motivation and will also affect the stability of the coalition.

At present, the study on the fuzzy preference of decision makers has achieved fruitful results [28,29,30,31]. However, there is little research on the uncertainty of preference statements. So, this paper takes into account the DMs’ uncertainty in preference statements based on option prioritization, and further measures the willingness and coalition stability. And the extended approach is applied to “the conflict of Changzhou Foreign Language School”.

The remainder of the paper is structured as follows: the next section introduces the framework of GMCR. An extended approach to design score function and real-coalition analysis is developed in Section 3. A case study is provided in Section 4. Conclusions are presented in the final section of the paper.

## 2. Framework of Graph Model for Conflict Resolution

The GMCR process includes modeling, stability analysis, and coalition analysis. The GMCR itself consists of four basic components: DMs (*N*), feasible states (*S*), preference (*P*), and possible state transitions (*G*). Each DM has its own options to choose or decide; *Y* indicates that the DM chooses the option, whereas *N* means that it does not. Assemble all options that have been selected by an individual DM as state s. Because not every state is achievable in the real world, the impossible condition is appointed as an infeasible state. The subset excluding unfeasible states is the feasible states subset *S*. Based on the GMCR framework, the concepts relating to subsets of states are summarized in Table 1. Term *P* represents a feasible preference state sequence; feasible states are ordered by user or a DM according the goal that the focal DM wants to achieve. Each DM has its own preference state sequence. Since it is difficult for a user to obtain complete DM preference information about an actual conflict event, preference is divided into four types: uncertainty preference, general preference, strong preference, and multiple preferences. Term *G* exhibits state transition graph, where a state transition means a DM unilaterally changes the option and shifts to another state.

Each DM is rational and wants to achieve the most preferred state for itself. Decision makers only make decisions about their own options, and are unable to intervene in those of others. The GMCR predicts the state that is most likely to be accepted by all DMs; this state is known as the equilibrium state. The course of solving for the equilibrium state is specified as stability analysis. There are four basic stability formations in GMCR: Nash stability (R), general meta-rationality (GMR), symmetric meta-rationality (SMR), and sequential stability (SEQ). If a stable state is reached by all DMs under a certain stability, then it is an equilibrium state for conflict analysis. However, not all equilibrium states can successfully solve conflict in the real world; we also need the user or DMs to judge and analyze realities.

Path analysis can be used to find all states that DMs need to transit from the status quo to the equilibrium state. There is often more than one path from the status quo to the equilibrium state; the transition state and the number of steps are frequently diverse. Hence, the meaning of path analysis is to find the shortest path, provide information for DMs to control the evolution of the conflict, improve the efficiency and quality of decision-making, and solve the conflict effectively. In the transition route to the equilibrium state, each DM can only transfer to the state that is preferred to its present state. In other words, transition states belong to the subset of states that DM*_i_* can reach unilaterally from state s. After achieving the equilibrium state, coalition analysis makes it necessary to identify whether the equilibrium state is still an equilibrium after considering cooperation. The purpose of coalition analysis is to identify the equilibriums that are vulnerable to the action of a coalition that could achieve another equilibrium preferred by all its members [16]. If the equilibrium state is not robust to the consideration of a coalition, then equilibrium jumps will happen. This means the equilibrium state is just temporary, and not a long-term equilibrium. 

## 3. Score Function and Real-Coalition Analysis

The discussion outlined in the next subsection concerns the specified score function and real-coalition analysis. The score function is used for quantify a DM’s state preference within the GMCR. Subsequently, real-coalition analysis is defined to enhance the applicability of the graph model.

### 3.1. Score Function

Denote C as a DM’s level of confidence in its judgment of a preference statement. A higher value of C implies that the preference statement is more suitable for all states, while a lower value of C indicates that it is less appropriate:

(1) $$C_{t}=\{\begin{array}{c} 1,\;t\leq p\\ {\left(\frac{q+1-t}{q+1-p}\right)}^{g},\;p+1\leq t\leq q\; \end{array},\;g\in \left\{1,2,\cdots ,n\right\},$$

There are q preference statements in total; p is the critical point in judging preference statements, and g is weight that decision maker consider the importance of the level of confidence. Form the first to p-th preference statements, the DMs are fully confident in their judgments on preference statements. Starting from the (p+1)-th preference statement, the certainty of the DMs’ judgment begins to weaken gradually.

**Definition** **1.** Function (1) expresses DM confidence in the t-th preference statement and is the appointed confidence level function.

To further highlight the priority weights of preference statements, one important task is to value the preference statements. The priority of preference statements is defined as follow, according to the “preference tree” idea put forward by Hipel.

**Definition** **2.** $\alpha^{t}$ *is defined to express the priority of preference statements.* $\left(\alpha >0,\;\alpha +\alpha^{2}+\alpha^{3}+\ldots +\alpha^{q}<1\right)$ *and* α∈[0,1].

Apparently, higher-priority preference statements are given greater weight, and lower-priority preference statements are given less weight.

In order to describe whether the state k of DM*_i_* complies with individual preference statements.

**Definition** **3.** *For all* k∈{1,2,3,⋯,n}, *if* $s_{k}$ *conforms with preference statement* $\Omega_{t}$, *then* $\sigma_{t}=1$; *if* $s_{k}$ *does not meet the preference statement* $\Omega_{t}$, *then* $\sigma_{t}=0$, *which can be represented in Equation (2)*.

(2) $$\sigma_{t}\left(\Omega_{t},S_{k}\right)=\{\begin{array}{c} 1,\mathrm{True};\;\\ 0,False;\; \end{array}$$

**Definition** **4.** *For all preference statements, score function* $SC\left(s_{k}\right)$ *is defined to characterize the DM’s score at a particular state* $s_{k}$, *as shown in Equation (3)*

(3) $$SC\left(s_{k}\right)=\sum_{t=1}^{q}[C_{t}{\left(\frac{1}{2}\right)}^{t}\sigma_{t}\left(\Omega_{t},S_{k}\right)],\;\mathrm{for}\;\mathrm{all}\;k=1,2\ldots n$$

Score $SC\left(s_{k}\right)$ can be calculated for each feasible state for a particular DM*_i_*. The states can be ranked according to their scores; a state with a higher score is preferred to a state with a lower score, more specifically for $s_{1},s_{2}\in S,\;s_{1}>s_{2}$ if and only if $SC\left(s_{1}\right)>\;SC\left(s_{2}\right)$.

### 3.2. Real-Coalition Analysis with Two DMs 

On the basis of calculating the score SC for each feasible state for a particular DM*_i_*, the differences in coalition improvement can be captured. We can then compare the coalition motivation of each DM and judge the strength of coalition stability. In this research, we consider a coalition with two DMs temporarily.

**Figure 1:**
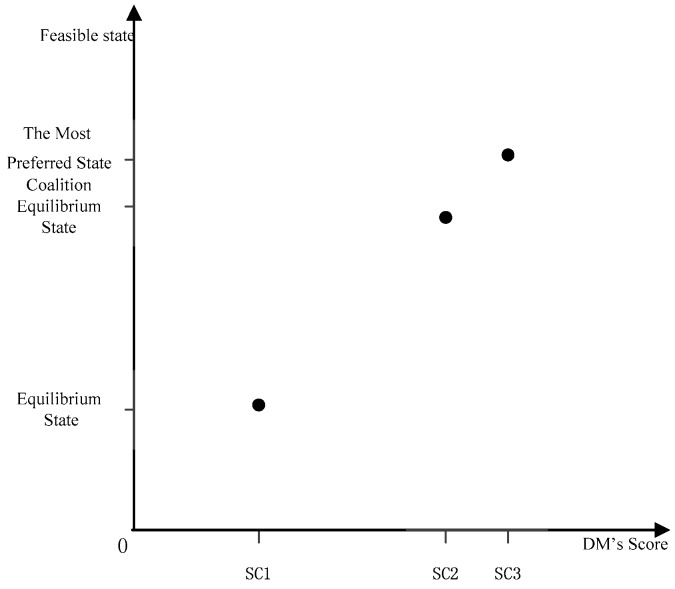
Decision Makers’ (DMs’) Score Coordinate System.

In order to measure coalition motivation, a two-dimensional coordinate system needs to be established to describe the reforming. We represent the equilibrium state, the coalition equilibrium state, and the most preferred state in the coordinate system, as well as the corresponding score SC of the three states. It is noticeable that the mapping relationship between states and scores is *f: x→y = x*. As illustrated in Figure 1, the horizontal axis means the score of DMs in different states, and the vertical axis represents states.

**Definition** **5.** *The degree of Collation Improvement, denoted by* CI *is given by Equation (4)*.

(4) $$CI=\{\begin{array}{l} \frac{{\mathrm{SC}}_{2}-{\mathrm{SC}}_{1}}{{\mathrm{SC}}_{3}-{\mathrm{SC}}_{2}},{\mathrm{SC}}_{2}\neq {\mathrm{SC}}_{3}\\ +\infty ,{\;\mathrm{SC}}_{2}={\mathrm{SC}}_{3} \end{array}\left({\mathrm{SC}}_{1}\neq {\mathrm{SC}}_{2},and\right)CI\geq 0$$

From the geometric meaning, the value of CI corresponds to the ratio of segment ${\mathrm{SC}}_{2}{\mathrm{SC}}_{3}$ and segment ${\mathrm{SC}}_{1}{\mathrm{SC}}_{2}$. For DM*_i_*, when CI=1, the coalition equilibrium state’s score ${\mathrm{SC}}_{2}$ is located in middle between ${\mathrm{SC}}_{1}$ and ${\mathrm{SC}}_{3}$. At this point, the improved degree is moderate. When CI<1, ${\mathrm{SC}}_{2}$ is closer to ${\mathrm{SC}}_{1}$ on the X axis, meaning that the coalition equilibrium state is closer to equilibrium state. In other words, the improved degree is not as good as expected. When CI>1, ${\mathrm{SC}}_{2}$ is closer to ${\mathrm{SC}}_{3}$ on the X axis, meaning that the coalition equilibrium state is closer to the most preferred state, i.e., the improved degree is more ideal. When CI=+∞, the coalition equilibrium state is equal to the most preferred state. This situation is the best result for coalition. In short, the degree of coalition improvement is positively correlated with the value of CI.

**Definition** **6.** *For any subset* H⊆N, *and* |H|=2*, if state s in* $S_{H}\left(s\right)$ *are those that can be attained from s by a move by a member of H, or by a sequence of moves by members of H, then members of H are defined as a coalition*.

**Definition** **7.** *A state s* ∈S *is coalition stable if and only if it is stable for all coalitions* H⊆N.

For DMs, the motivated coalition identifies that all members of coalition H prefer the target state to the status quo [29]. So we have sufficient reason to weight the coalition motivation by a coalition member’s CI that has been discussed above. Therefore, we can judge each DM’s willingness and accurately describe the tendency of coalition formation, namely which DM will be more active.

For |H|=2, a DM with a higher CI corresponds to a stronger coalition motivation, that is, a greater willingness to form a coalition. In the process of forming a coalition, that DM will take the initiative to communicate and convey the desire of coalition formation or take corresponding strategies to facilitate coalition. 

Next, we will discuss real-coalition stability. If equilibrium jumps exist, then the decision support system GMCR II will disseminate the outcome of coalition. However, in the real world, because of external circumstances as well as the opportunity cost in the practice of coalition, DMs may not be successful in forming a coalition. Even if the DMs can form a coalition, the interference of external circumstances and non-coalition DMs or outsiders will possibly lead to the breakup of the coalition relationship. In this section, we attempt to construct a paradigm based on the value of CI to measure the possibility of coalition and the coalition relationship stability, and to make GMCR more in line with the real world and have practical significance. The possibility and coalition relationship stability that consider the external environment and the opportunity cost are defined as real-coalition stability.

If the values of CI for both coalition decisions are less than 1, i.e., $CI\left(H_{1}\right)<1$ and $CI\left(H_{2}\right)<1$, then the improvement degree is not good. Hence, in the process of coalescing, the two sides will conduct consultations and negotiations many times with high opportunity costs, thus sharply reducing the possibility of forming a coalition. Moreover, this situation can easily lead to coalition relations rupturing or a coalition being unable to evolve because of the obstruction of external circumstances and the temptation of interests by non-coalition DMs or other outsiders. Thus, the coalition relationship will break up effortlessly; hence, the real-coalition stability is poor. 

Now suppose that the values of CI for both coalition decisions are greater than 1, i.e., $CI\left(H_{1}\right)>1$ and $CI\left(H_{2}\right)>1$ In that case, the real-coalition stability is steady and the degree of improvement is very significant. Hence, both sides have a strong will to coalesce, and the coalition status will not be interrupted by external conditions or other outsiders. In other words, the coalition relationship is tight. If the CI value of one of two co-DMs is less than 1 or both are equal to 1, the real-coalition stability is moderate.

## 4. Application of Improved GMCR

### 4.1. Background of Conflict and Modeling

With the purpose of improving its air quality, Changzhou New District launched the “Chang Long Chemical” block chemical-industry seat relocation in May 2009, and formally implemented land restoration in March 2014. The Changzhou Foreign Language School moved to a new campus adjacent to the repaired block in September 2015; by the end of 2015, many of its students were in physical discomfort. On 17 April 2016, the CCTV news channel reported the event headlined “Shouldn’t Build School”, in which nearly 500 Changzhou Foreign Language School students showed signs of physical discomfort. The suspected reason was that the school had been relocated close to the original “Chang Long Chemical” industrial land. Individual students were even suffering from leukemia, lymphoma, and other malignant diseases.

Experts said that the reason for the symptoms of discomfort was that the restoration operation had not met specifications. The Black Peony Company was responsible for the land reparation project. Its actual restoration method of “cover on spot” was not in accordance with the specified method of “completely closed”, which resulted in the spread of pollution of volatile organic pollutants in the soil. After the accident had been reported, the Government of Changzhou organized experts to design and carry out an emergency plan and an adjustment scheme. The event incited multiple conflicts. In this study, we pay particular attention to the conflict that arose in relation to the health problems of teachers and students at the Changzhou Foreign Language School (hereinafter called the “Changzhou Conflict”).

The Environmental Protection Agency hoped to solve the environmental pollution problem fundamentally. The Black Peony Company pursued revenue maximization of its enterprises. The Government of Changzhou wanted to maximize the social and economic benefits under the premise of ensuring the physical health of its teachers and students. The date selected for the GMCR II analysis was 27 April 2016.

DMs and Options the—above-mentioned Changzhou Conflict was mainly related to three DMs: the Environmental Protection Agency (EPA), the Black Peony Company (BPC), and the Government of Changzhou (GC). The options of each DM are listed in Table 2.

In the graph model of the Changzhou Conflict, there are 64 states in theory. However, some of the states are logically infeasible; for instance, BPC cannot choose the two options of “completely closed” method and “cover on spot” method simultaneously, nor can they choose neither option. Excluding infeasible states, 12 types of feasible state ultimately remain, as listed in Table 3. For example, State 1 indicates that BPC chooses the “completely closed” method to purify the contaminated land, and GC decides to relocate the school site temporarily.

**Figure 2:**
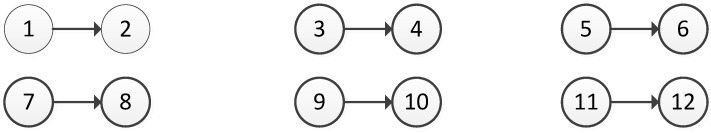
Graph Model of Changzhou Conflict for Movement by EPA.

**Figure 3:**
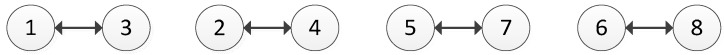
Graph Model of Changzhou Conflict for Movement by BPC.

**Figure 4:**
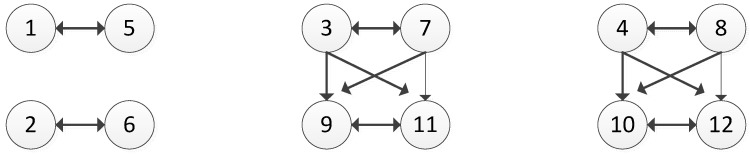
Graph Model of Changzhou Conflict for Movement by GC.

Figure 2, Figure 3 and Figure 4 exhibit the graph model of the Changzhou Conflict in terms of movement by EPA, BPC, and GC, respectively.

In this study, we use option prioritization to order the states; we get preference state sequences according to preference statements. A positive number (+) indicates that the DMs prefer the preference statement; “—” express that DMs hope it will not happen; “&” represents “and”, i.e., the intersection of two options; “|” represents “or”; IF means “if”…; and IFF signifies “if and only if”. A term such as 6IF3 denotes “IF BPC remain with covering on spot, then EPA hopes that GC will punish BPC”. The preference statements of EPA are represented vertically in Table 4 from the most preferred to the least preferred. The EPA desires most that BPC will comply strictly with the operational specifications. Their second preference is to monitor BPC as it purifies the contaminated land.

DM1 (Environmental Protection Agency): P=[2>6>1>5>10>12>4>8>9>11>3>7]. Based on the preference statements of EPA listed in Table 4, the decision support system GMCRII gives out the preference state sequence of EPA. Among them, the left-most-ranked state represents the DMs’ most preferred state, whereas the right-most-ranked state signifies the least preferred state. For instance, EPA’s strongest preference is for BPC to adopt the “completely closed” approach to purify the polluted soil, GC to decide to relocate the school site, and EPA itself to oversee the practice of curing the polluted soil. The least-preferred option is for BPC to choose the “cover on spot” approach, that is, GC to punish the company and not move the school. Similarly, the preference statements of BPC and GC are expressed in Table 5 and Table 6, respectively, and the corresponding preference state sequences are as given by GMCR II.

DM2 (Black Peony Company): P=[3>7>4>8>1>5>2>6>9>11>10>12]. DM3 (The Government of Changzhou): P=[6>2>5>1>12>10>8>4>11>9>7>3].

Next, we consider the preference state sequences provided by the means of developed option prioritization. In this case, g=2; $\alpha =\frac{1}{2}$, and $\alpha^{t}={\left(\frac{1}{2}\right)}^{t}$. Table 7 shows the three DMs’ levels of confidence in their judgments.

Each state score for an individual DM can be calculated on the basis of the score function discussed above, and is displayed in Table 8, Table 9 and Table 10.

The ranked states according to SC for all DMs are summarized as follows:

P(DM1)=[2>6>1>5>10>12>4>8>9>11>3>7];P(DM2)=[3>7>4>8>1>5>2>6>9>11>10>12]′P(DM3)=[6>2>5>1>12>10>8>4>11>9>7>3];

The new preference state sequences are consistent with the original results that relied on option prioritization.

### 4.2. Stability Analysis

According to the definitions of the four stabilities, the equilibrium states can be drawn as shown in Table 11, in which indicates that the state is an equilibrium state corresponding to the respective stability definition.

For Nash stability, the equilibrium state is State 12. For GMR, SMR, and SEQ stabilities, the equilibrium states are 1, 2, 5, 6, and 12. State 12 is a strong equilibrium, but it cannot fundamentally resolve the conflict. State 12 implies that EPA is involved in and supervises BPC, but BPC still chooses the “cover on spot” approach to purify the soil, which not only cannot cure the environmental problem but is also a hazard to the health of the teachers and students. Based on these, GC penalizes BPC and relocates the school site. This is obviously a far-from-ideal solution to solving the Changzhou Conflict. State 6 indicates that under the supervision of EPA, BPC adopts the “completely closed” means of purifying the contaminated land, which would reduce the impact of volatile pollutants on teachers and students. Under the premise of effectively protecting the health of students and teachers, GC does not relocate the school site, and this state would effectively coordinate the interests of all DMs. State 2 shows EPA being involved in overseeing the company’s purification course and BPC choosing the “completely closed” method, while GC still decides to relocate the school site. Comparing States 2 and 6, the latter is a better solution based on the consideration of social and economic benefits.

**Figure 5:**
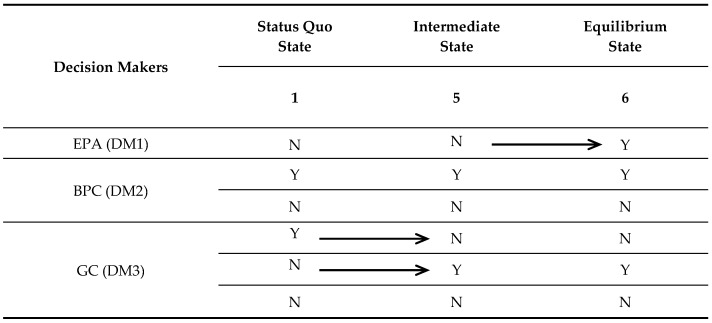
Evolution of the conflict from the status quo through an intermediate state to the final resolution.

In the case of the Changzhou Conflict discussed above, Figure 5 describes the path of the conflict from the status quo through an intermediate state to the final resolution. After BPC purifies the soil by fully enclosing the site, GC decides not to move the Changzhou Foreign Language School (move from State 1 to State 5). In order to actually protect the teachers and students, and to prevent a similar accident from happening again, EPA decides to supervise BPC. This state not only achieves the resolution of healing the environment properly, but also diminishes the physical harm to teachers and students. At this point, each participant in the Changzhou Conflict reaches a balanced state.

### 4.3. Coalition Analysis

**Figure 6:**
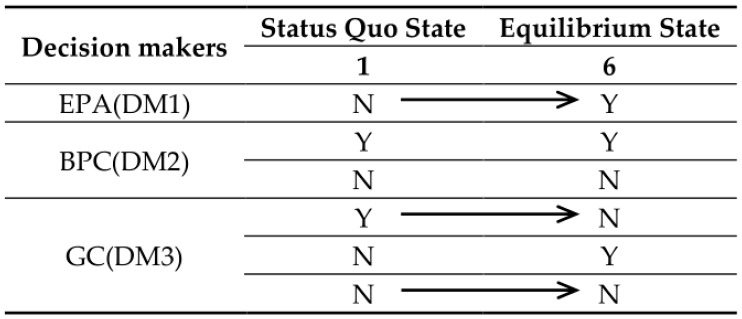
Evolution of Changzhou Conflict from the Status Quo to the Final Resolution.

Comparing Table 11 and Table 12, we find the following: States 1 and 5 are balanced, but while the DMs consider a coalition, States 1 and 5 are not in equilibrium. Now, suppose that there are equilibrium jumps after the DMs reach a coalition. Figure 6 draws the path of an equilibrium jump from State 1 to State 6. The GC contracts an alliance with EPA: with the support of GC, EPA takes action to supervise BPC, binding upon it. On this occasion, having effectively protected the health of the teachers and students, GC decides not to relocate the school site on the basis of the social and economic benefits. In other words, there is a direct transfer from State 1 to State 6.

The main reasons for coalition as far as GC and EPA are concerned are that State 6 is better than State 1 for both coalition participants, and neither can get to State 6 by unilateral improvement. From a practical point of view, it is very common for government offices to partner in the case of an emergency.

### 4.4. Coalition Motivation and Real-Coalition Stability Analysis

**Figure 7:**
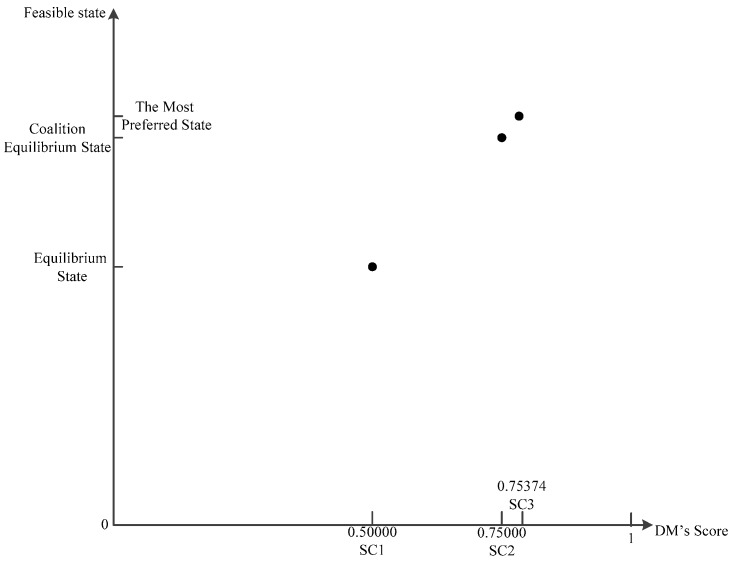
Score coordinate system of EPA (DM1).

**Figure 8:**
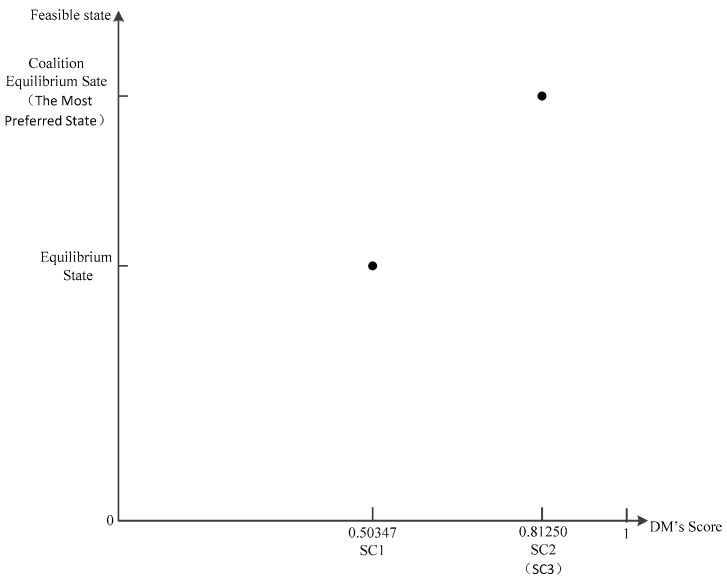
Score Coordinate System of GC (DM3).

In line with the values of SC or EPA and GC, we establish the appropriate score coordinate system as depicted in Figure 7 and Figure 8.

$CI\left(H_{1}\right)=7.205$. Because CI>1, we say that the state improved significantly with a coalition between GC and EPA. As shown in Figure 7, points $SC_{2}$ and $SC_{3}$ are relatively close together. The SC of the coalition equilibrium state is closer to the SC of the most preferred state.

$CI\left(H_{2}\right)=+\infty$; hence, the coalition equilibrium state is the most preferred state, which is the best result for a coalition. After coalition, the states of EPA and GC improve significantly, but the level of improvement for GC is evidently better than that for EPA. So, as the coalition progresses, GC will be more motivated to promote the coalition and devote itself to contribute to it. The values of CI for both coalition DMs are greater than 1, i.e., $CI\left(H_{1}\right)>1$ and $CI\left(H_{2}\right)>1$. This implies that both parties would find it easy to reach a consensus in the course of the coalition. The coalition relationship would be noticeably solid, and the coalition status would not be disturbed by exterior circumstances. In reality, coalitions between government departments tend to be exceedingly tight, so this finding is more consistent with real life.

## 5. Conclusions

Having considered the difference in the state preferences of DMs, we proposed a confidence level function, $\alpha^{t}$, and a Boolean function about preference statements as a foundation on which to construct a score function We then proposed a paradigm for measuring the coalition motivation of each DM, and further studied real-coalition stability. Finally, we applied an improved GMCR to the Changzhou Conflict, contrasting the original and new methods to find that the new preference state sequences were consistent with the original results. Through real-coalition stability analysis, we found the following: The Government of Changzhou would be more autonomous and would commit itself to coalition, and an alliance with the EPA would not be interrupted by external factors or other outsiders, meaning that the coalition relationship would not be easily broken up. Future research could be expanded from the following two aspects: (1) identify the main contradiction through sensitivity analysis to more effectively address the problem or guide the evolution of a conflict toward an expected direction; and (2) accurately measure the impact of opportunity cost on coalition results in the real world by a quantitative method.

## Figures and Tables

**Table 1:** Concepts of subsets of states.

Subset of States	Implication
$R_{i}\left(s\right)$	The subset of states that DM*_i_* can unilaterally reach from state s.
$R^{+}{}_{i}\left(s\right)$	The subset of states that DM*_i_* preferred to state s and can unilaterally reach from state s.
$R^{=}{}_{i}\left(s\right)$	The subset of states that is equivalent to state s and can unilaterally reach from state s.
$R_{H}\left(s\right)$	The subset of states that coalition decision maker H can unilaterally reach from state s.
$R^{+}{}_{H}\left(s\right)$	The subset of states that all coalition decision maker H preferred to state s and can unilaterally reach from state s.

DM: decision maker.

**Table 2:** DMs and options in the Changzhou conflict.

Decision Makers (DMs)	Options
EPA (DM1)	1. Supervise the process of field repair.
BPC (DM2)	2. Comply with specification: “completely closed” method.
	3. Maintain present status: “cover on spot” method.
GC (DM3)	4. Relocate school site temporarily.
	5. Don’t relocate school site.
	6. Punish BPC.

Note: EPA: the Environmental Protection Agency; BPC: the Black Peony Company; GC: the Government of Changzhou.

**Table 3:** Feasible states of Changzhou conflict.

Decision Makers	Options	Feasible States											
		1	2	3	4	5	6	7	8	9	10	11	12
EPA (DM1)	Supervise	N	Y	N	Y	N	Y	N	Y	N	Y	N	Y
BPC (DM2)	“Completely closed”	Y	Y	N	N	Y	Y	N	N	N	N	N	N
	“Cover on spot”	N	N	Y	Y	N	N	Y	Y	Y	Y	Y	Y
GC (DM3)	Relocate	Y	Y	Y	Y	N	N	N	N	Y	Y	N	N
	Don’t relocate	N	N	N	N	Y	Y	Y	Y	N	N	Y	Y
	Punish	N	N	N	N	N	N	N	N	Y	Y	Y	Y

**Table 4:** Preference statements of EPA.

Interpretation	Preference Statements
BPC complies with operational specifications.	−3|2
Supervise the process of field repair.	1
If BPC retains coverage on the spot, then EPA hopes that GC will punish BPC.	6IF3
If BPC retains coverage on spot, then EPA hopes that GC will relocate school site temporarily.	4IF3
Relocate school site temporarily.	4

**Table 5:** Preference statements of BPC.

Interpretation	Preference Statements
Don’t want to be penalized by GC.	−6
Continue choosing “cover on spot” method.	3
Don’t want EPA to be involved.	−1
Relocate school site temporarily.	4

**Table 6:** Preference Statements of GC.

Interpretation	Preference statements
Hope BPC complies with specifications.	−3|2
EPA supervises the process.	1
If EPA supervises while BPC continues covering on spot, then GC will penalize BPC.	6IF(1 and 3)
Don’t relocate school site.	5

**Table 7:** C of Preference Statements of DM1, DM2 and DM3.

Serial Number of Preference Statement	DM1	*C*1	DM2	*C*2		DM3	*C*3	
1	*p* = 3	1	*p* = 3	1		*p* = 4	1	
2		1		1			1	
3		1		1			1	
4		4/9		1/4			1	
5		1/9						
			

**Table 8:** SC of EPA.

**Decision Maker**	**State**	**1**	**2**	**3**	**4**	**5**	**6**
	SC	0.50347	0.75347	0.02778	0.25010	0.50000	0.75000
**DM1**	**State**	**7**	**8**	**9**	**10**	**11**	**12**
	SC	0.00000	0.25000	0.15625	0.40625	0.12500	0.37500

**Table 9:** SC of BPC.

**Decision Maker**	**State**	**1**	**2**	**3**	**4**	**5**	**6**
	SC	0.64063	0.51563	0.89063	0.76563	0.62500	0.50000
**DM2**	**State**	**7**	**8**	**9**	**10**	**11**	**12**
	SC	0.87500	0.75000	0.39063	0.26563	0.37500	0.25000

**Table 10:** SC of GC.

**Decision Maker**	**State**	**1**	**2**	**3**	**4**	**5**	**6**
	SC	0.50000	0.75000	0.00000	0.25000	0.56250	0.81250
**DM3**	**State**	**7**	**8**	**9**	**10**	**11**	**12**
	SC	0.06250	0.31250	0.12500	0.37500	0.18750	0.43750

**Table 11:** Equilibrium states of the Changzhou conflict.

Stability	Equilibrium States				
	1	2	5	6	12
Nash					✓
GMR	✓	✓	✓	✓	✓
SMR	✓	✓	✓	✓	✓
SEQ	✓	✓	✓	✓	✓

GMR: general meta-rationality; SMR: symmetric meta-rationality; SEQ: sequential stability.

**Table 12:** Coalition equilibrium states of Changzhou conflict.

Stability	Coalition Equilibrium States		
	2	6	12
Nash			✓
GMR	✓	✓	✓
SMR	✓	✓	✓
SEQ	✓	✓	✓
